# Uptake and cytotoxicity of citrate-coated gold nanospheres: Comparative studies on human endothelial and epithelial cells

**DOI:** 10.1186/1743-8977-9-23

**Published:** 2012-07-03

**Authors:** Christian Freese, Chiara Uboldi, Matthew I Gibson, Ronald E Unger, Babette B Weksler, Ignacio A Romero, Pierre-Olivier Couraud, C James Kirkpatrick

**Affiliations:** 1 REPAIR-lab, Institute of Pathology, University Medical Center of the Johannes Gutenberg University, Langenbeckstraße 1, Mainz, 55101, Germany; 2 European Institute of Excellence on Tissue Engineering and Regenerative Medicine, Mainz, Germany; 3 University of Warwick, Department of Chemistry, Coventry, UK; 4 Institut Cochin, Centre National de la Recherche Scientifique UMR 8104, Institut National de la Santé et de la Recherche Médicale (INSERM) U567, Université René Descartes, Paris, France; 5 Weill Medical College of Cornell University, New York, NY, 10021, USA; 6 Department of Biological Sciences, The Open University, Walton Hall, Milton Keynes, UK; 7 Joint Research Centre of the European Commission, IHCP Nanobiosciences Unit, Ispra, Italy

## Abstract

**Background:**

The use of gold nanoparticles (AuNPs) for diagnostic applications and for drug and gene-delivery is currently under intensive investigation. For such applications, biocompatibility and the absence of cytotoxicity of AuNPs is essential. Although generally considered as highly biocompatible, previous in vitro studies have shown that cytotoxicity of AuNPs in certain human epithelial cells was observed. In particular, the degree of purification of AuNPs (presence of sodium citrate residues on the particles) was shown to affect the proliferation and induce cytotoxicity in these cells. To expand these studies, we have examined if the effects are related to nanoparticle size (10, 11 nm, 25 nm), to the presence of sodium citrate on the particles' surface or they are due to a varying degree of internalization of the AuNPs. Since two cell types are present in the major barriers to the outside in the human body, we have also included endothelial cells from the vasculature and blood brain barrier.

**Results:**

Transmission electron microscopy demonstrates that the internalized gold nanoparticles are located within vesicles. Increased cytotoxicity was observed after exposure to AuNPs and was found to be concentration-dependent. In addition, cell viability and the proliferation of both endothelial cells decreased after exposure to gold nanoparticles, especially at high concentrations. Moreover, in contrast to the size of the particles (10 nm, 11 nm, 25 nm), the presence of sodium citrate on the nanoparticle surface appeared to enhance these effects. The effects on microvascular endothelial cells from blood vessels were slightly enhanced compared to the effects on brain-derived endothelial cells. A quantification of AuNPs within cells by ICP-AES showed that epithelial cells internalized a higher quantity of AuNPs compared to endothelial cells and that the quantity of uptake is not correlated with the amount of sodium citrate on the nanoparticles’ surface.

**Conclusions:**

In conclusion the higher amount of citrate on the particle surface resulted in a higher impairment of cell viability, but did not enhance or reduce the uptake behavior in endothelial or epithelial cells. In addition, epithelial and endothelial cells exhibited different uptake behaviors for citrate-stabilized gold nanoparticles, which might be related to different interactions occurring at the nanoparticle-cell-surface interface. The different uptake in epithelial cells might explain the higher reduction of proliferation of these cells after exposure to AuNPs treatment although more detailed investigations are necessary to determine subcellular events. Nevertheless an extrinsic effect of sodium-citrate stabilized particles could not be excluded. Thus, the amount of sodium citrate should be reduced to a level on which the stability of the particles and the safety for biomedical applications are guaranteed.

## Background

Interest in using gold nanoparticles (AuNPs) in medical applications has surged in the last few years following numerous studies showing that drug, protein or gene delivery and cancer therapies, imaging enhancements, as well as further medical diagnostics are more efficient when coupled to nanoparticles [1,2]. However, with the increase in the production of unique nanomaterials a need arises for well-defined methods to evaluate and characterize nanomaterials in order to ensure that safety risks for people and the environment are as low as possible.

Although AuNPs appear to be promising for medical applications on account of their bioinert character and high biocompatibility, several studies have shown that a toxic effect of AuNPs on cells cannot be excluded. AuNPs can cause nephrotoxicity and cell death of erythrocytes *in vivo*[3,4] and 13 nm-sized PEGylated gold nanoparticles were reported to accumulate in the liver and to induce inflammation and apoptosis in BALB/c mice [5]. In addition to time- and dose-dependent effects of AuNPs, physicochemical properties, such as surface charge and surface modification as well as shape and size of the nanoparticles, may play a role in producing toxic effects, and influence their aggregation state [6,7]. In addition, it has been shown that the surface charge of AuNPs highly influences the uptake properties in ovarian cancer CP70 cells [8]. Compared to anionic gold nanoparticles, cationic gold nanoparticles have a higher affinity for the negatively-charged cell surface residues. However, the positive charges of the surface-modified nanoparticles can also result in an increased cytotoxicity in HeLa [9], ovarian cancer cells CP70, A2780 and the airway cells, BEC and ASM [8]. These studies revealed that although the bulk material is bioinert, the nano-sized counterparts can lead to cytotoxicity due to the altered surface properties which accompany decreased size. Recently, we have also shown that the presence of stabilizers, such as sodium citrate, on the surface of gold nanoparticles can induce cytotoxicity in the human lung AT-II-like cell lines, A549 and NCIH441 [10].

Epithelial cells are the primary cell types that have been used to examine the toxicological and mechanisms of uptake in *in vitro* studies. Endothelial cells, which are the primary cell-type making up the vasculature, are present in nearly all tissues and organs, and are primarily responsible for the transport of nutrients and waste, as well as presenting a barrier to the infiltration of bacteria and viruses as well as toxic compounds and molecules. Investigations with endothelial cells and their interaction with nanoparticles are necessary since these cells will most likely come into contact with nanoparticles after exposure, whether absorption via the skin or mucosal surfaces, inhalation, swallowing, or injection. The key role played by vascular endothelial cells in physiological and pathological processes [11,12] makes study of the effects of nanoparticles on endothelial cells of great interest and relevance for clinical applications. Moreover, nanoparticulate drug-delivery systems need to overcome the barrier generated by endothelial cells to reach the region of interest. Thus, the uptake, transport and possible accumulation of nanoparticles in endothelial cells need to be considered, especially with respect to the heterogeneity of the endothelium. To further investigate the effects that gold nanoparticles have on human cells, in these studies we have examined human endothelial cells to compare and to determine if differences in the amount of internalized nanoparticles in epithelial and endothelial cells exist. In addition to the investigations with human cell lines, Kunzman and co-workers recommended the use of primary cell cultures as model systems. Primary cultures, in fact, simulate more closely the *in vivo* situation than immortalized cell lines, so that the comparison between primary and immortalized cells is of great importance in establishing suitable models to investigate cytotoxic effects [13]. The need to study endothelial cell interaction with nanoparticles is also demonstrated by the recent literature. The group around Kanaras investigated the interaction between primary macrovascular human endothelial cells (Huvec) and gold nanoparticles with different morphologies [14,15].

The goal of the present studies was to examine the effects of gold nanoparticles on the viability of human microvascular endothelial cells, whereby comparisons were made between primary human dermal microvascular endothelial cells (HDMEC) and a human cerebral microvascular endothelial cell line (hCMEC/D3). We also investigated the relationship between cytotoxicity, the amount of internalized AuNPs and if the presence of gold nanoparticle stabilizers, such as sodium citrate, affects the viability and the uptake of gold nanoparticles in HDMEC and hCMEC. To determine this we quantified and compared the amount of internalized gold nanoparticles in endothelial cells using inductively coupled plasma-atomic emission spectroscopy (ICP-AES) and compared the results to those observed in epithelial cell lines (A549 and NCIH441).

## Results

### Cytotoxicity of gold nanoparticles on endothelial cells

**Figure 1:**
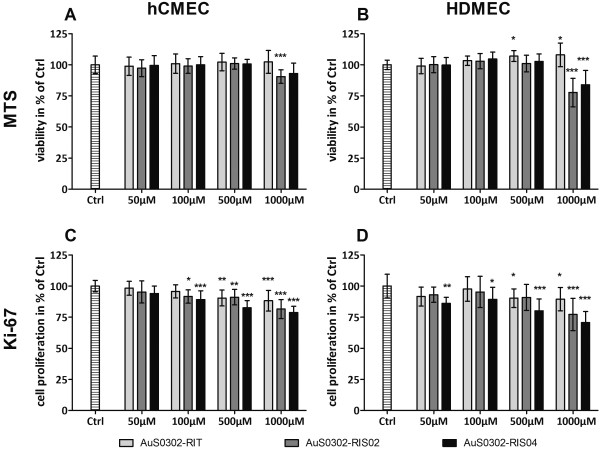
**Viability and proliferation of endothelial cells after 48 h exposure to AuNPs.** The endothelial cells hCMEC/D3 (**A** + **C**) and HDMEC (**B** + **D**) were exposed for 48 hours to different amounts of gold nanoparticles ranging from 50 μM up to 1000 μM. Cell viability was measured by the MTS assay (**A** + **B**) and compared to untreated cells (Ctrl), whose viability was set at 100%. The proliferation was detected by measuring the proliferation factor Ki-67 (**C** + **D**). Each result represents the mean ± standard deviation of four independent experiments, each of these was performed at least in triplicates (ONEway ANOVA with Dunnetts t-test: * P < 0,5; ** P < 0,05; *** P < 0.001).

To determine if 10 nm- and 25 nm-sized gold nanoparticles (AuNPs) exhibit cytotoxic effects on HDMEC and hCMEC/D3, cells were exposed to different concentrations (50-100-500-1000 μM) of AuNPs for 48 hours and cell viability was measured using the CellTiter 96® AQueous Non-Radioactive Cell Proliferation Assay (MTS) (Figure 1). A decrease in the cell viability of hCMEC (Figure 1A) was only observed when AuNPs concentrations were above 500 μM. Only a slight decrease in viability was observed after exposure to 1000 μM AuS0302-RIS02 and AuS0302-RIS04. In contrast to the AuNPs with an excess of sodium citrate on their surface (AuS0302-RIS02 and AuS0302-RIS04), no effect on the viability of hCMEC/D3 was observed after 48 hours of AuS0302-RIT exposure. In addition, HDMEC (Figure 1B) were not negatively affected by exposure to 1000 μM AuS0302-RIT. However, a significant increase in cell viability to 107% and 108% compared to the untreated control was determined after the treatment with 500 μM and 1000 μM AuS0302-RIT, respectively. Interestingly, the cell viability of HDMEC (Figure 1B) significantly decreased after 48 hours of exposure to 1000 μM AuS0302-RIS02 and AuS0302-RIS04.

To identify a possible impact of AuNPs on the proliferation of HDMEC and hCMEC, the amount of nuclear Ki-67, a protein expressed by all cells in the active cell cycle, was determined after exposure to gold nanoparticles. In both HDMEC and hCMEC a dose-dependent decrease in the Ki-67 expression could be detected (Figure 1C and D). At low doses (50 μM and 100 μM) AuNPs slightly decreased the expression of Ki-67, while in HDMEC the treatment with 50 μM AuS0302-RIT04 significantly reduced the Ki-67 expression. In addition, after exposure to 500 μM of AuS0302-RIS04 a significant greater decrease of Ki-67 expression was observed. After exposure to high doses of 1000 μM AuS0302-RIS04 the expression further decreased. The smaller gold nanoparticles AuS0302-RIS02 (11 nm) induced a milder, but significant reduction of Ki-67 expression in both cell types compared to AuS0302-RIS04 (25 nm). After exposure to 1000 μM AuS0302-RIS02 the expression of Ki-67 was significantly impaired, while the expression of Ki-67 after exposure to 1000 μM AuS0302-RIT was only slightly decreased in both endothelial cell types.

**Figure 2:**
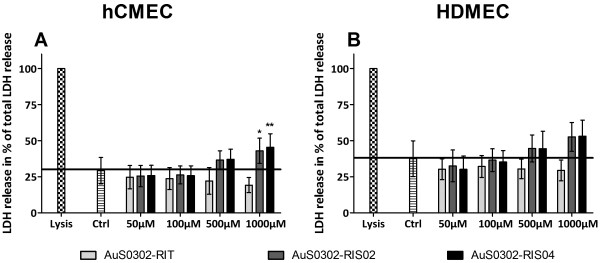
**LDH release in hCMEC/D3 and HDMEC after 48 h exposure to AuNPs.** The endothelial cells hCMEC/D3 (**A**) and HDMEC (**B**) were exposed to different amounts of gold nanoparticles (50 μM – 1000 μM) for 48 hours. Gold-induced cytotoxicity was measured by the LDH release into the supernatant by an enzymatic assay system. As positive control lysed cells were used and set at 100% (lysis). The spontaneous LDH releases of untreated cells are indicated by the black horizontal line. Each result represents the mean ± standard deviation of three independent experiments, each of these was performed at least in triplicates (ONEway ANOVA with Dunnetts t-test: * P < 0.5; ** P < 0.05; *** P < 0.001).

The induction of cytotoxicity after incubation with gold nanoparticles was further investigated by examining the amount of lactate dehydrogenase (LDH) released into the supernatant. Up to 100 μM gold nanoparticles did not induce cytotoxicity in HDMEC and hCMEC. Nevertheless, as shown in Figure 2, a concentration-dependent release of LDH following exposure to gold nanoparticles could be measured. Furthermore, AuS0302-RIT did not induce toxicity in HDMEC and hCMEC even after 48 h incubation. In contrast, compared to untreated control cells, a slight increase of LDH release into the supernatant was detected after stimulation of HDMEC and hCMEC/D3 with 500 μM and 1000 μM AuS0302-RIS02 and AuS0302-RIS04.

### Transmission electron microscopical analysis of the uptake of gold nanoparticles by different endothelial cell types

**Figure 3:**
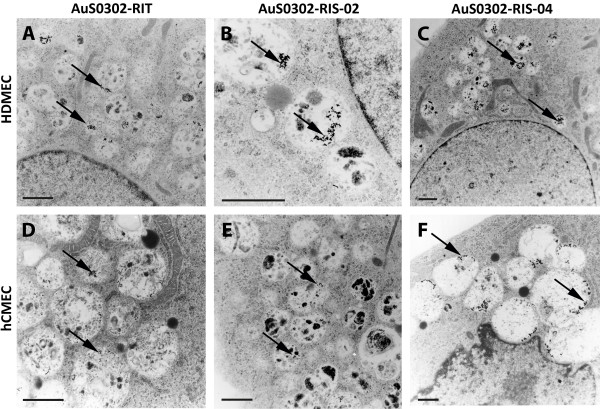
**Internalization of gold nanoparticles in HDMEC and hCMEC/D3 analyzed by transmission electron microscopy.** HDMEC (**A**-**C**) and hCMEC/D3 (**D**-**F**) were incubated with 300 μM gold nanoparticles for 24 hours. After exposure, cells were extensively washed, fixed with paraformaldehyde and examined by transmission electron microscopy (TEM). AuS0302-RIT, AuS0302-RIS02 and AuS0302-RIS04 were found in intracellular vesicles which were mostly located in the perinuclear region. The arrow heads indicate the gold nanoparticles within the vesicles. Scale bar: 1 μm.

To investigate the uptake of AuS0302-RIT, AuS0302-RIS02 and AuS0302-RIS04, we incubated HDMEC and hCMEC with a non-toxic dose of gold nanoparticles (300 μM) for 24 hours and then confirmed the uptake by transmission electron microscopical (TEM) analysis. In a previous study we demonstrated that AuS0302-RIT, AuS0302-RIS02 and AuS0302-RIS04 were taken up by the human alveolar type-II -like epithelial cell lines, A549 and NCIH441, after 3 hours incubation [10]. In the present study we investigated the quantity of internalized gold nanoparticles (AuS0302-RIT, AuS0302-RIS02 and AuS0302-RIS04) after an incubation time of 24 hours (see Additional file 1: Internalization of gold nanoparticles in A549 and NCIH441 analysed by transmission electron microscopy). As already shown in epithelial cells [10], it could be demonstrated that the gold nanoparticles in the same concentration were internalized by endothelial cells and were stored in vesicles mostly located in the perinuclear region (Figure 3). Additionally, no particles could be detected in the cytosol or in other cellular compartments, such as nuclei or mitochondria. Although a high concentration of AuNPs (300 μM) was used to investigate the uptake of AuS0302-RIT, AuS0302-RIS02 and AuS0302-RIS04 in HDMEC and hCMEC, only few nanoparticles were internalized compared to the reaction in epithelial cells [10]. In addition, the comparison of the amount of internalized gold nanoparticles did not reveal any difference that could be linked to the presence of different amounts of surface-bound sodium citrate or nanoparticle size. Moreover, differences in the amount of gold nanoparticles taken up into the two different endothelial cell types were not detected by TEM (Figure 3). To quantify the exact number of internalized gold nanoparticles, the entire cell population exposed to the nanoparticles was analyzed by inductively coupled plasma and atomic emission spectroscopy (ICP-AES).

### Quantification of the internalized gold nanoparticles

**Figure 4:**
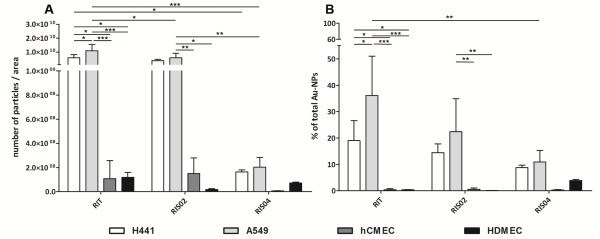
**Quantification of internalized gold nanoparticles in endothelial and epithelial cells by ICP-AES.** Both epithelial cells (H441 and A549) and endothelial cells (HDMEC and hCMEC/D3) were incubated with 50 μM gold nanoparticles at 37°C for 24 hours. Cells were extensively washed, lysed by aqua regia (3:1 hydrochloric acid : nitric acid) and analyzed for gold concentration by ICP-AES. In (A) the total number of particles per area was calculated, while in (B) the percentage uptake of particles into cells, as a function of the total amount applied, was determined. (duplicate of three independent experiments).

Compared to image analysis, inductively coupled plasma and atomic emission spectroscopy (ICP-AES) is a more sensitive and accurate method to quantify small amounts of gold nanoparticles within cells. ICP-AES can detect differences in the internalized gold concentrations of the order of parts per billion. In addition to the high sensitivity of the method a high number of cells can be analyzed simultaneously [15-18]. We analyzed the ICP-AES data in two different ways. In Figure 4A the number of particles was determined while in Figure 4B the percentage uptake of particles into cells, as a function of the initial amount applied, is shown. Both data sets are of importance since the nanoparticles which are not internalized might show a different biodistribution and cause side effects after application in vivo.

In general, compared to the endothelial cells, HDMEC and hCMEC, epithelial cells (A549 and NCIH441) internalized a larger number of all gold nanoparticles. Moreover, as demonstrated in Figure 4B the endothelial cells internalize a very small amount of gold nanoparticles compared to the initial amount applied (less than 5%). In addition to that, it was shown that a difference existed in the uptake of the smaller sized gold nanoparticles (AuS0302-RIT (10 nm), AuS0302-RIS02 (11 nm)) compared to AuS0302-RIS04 (25 nm), while the smaller particles were internalized in a higher quantity by both epithelial cells, A549 and NCIH441 (Figure 4A). Furthermore, the amount of particles internalized compared to that in the suspension is also less after treatment with AuS0302-RIS04 compared to AuS0302-RIT and –RIS02. However, a significant difference in internalized amounts of AuS0302-RIT and –RIS02 did not exist (Figure 4B), although the number of AuS0302-RIS02 compared to –RIT in A549 is significantly lower (Figure 4A). In contrast to epithelial cells and summarizing the result of both analyzes, HDMEC internalized AuS0302-RIS04 (25 nm) particles best but also in a lower quantity than the epithelial cells. However, the same uptake behavior was not detected in brain endothelial cells (hCMEC). Furthermore, a difference in the amount of internalized gold nanoparticles between the two epithelial cell types was also detected. The most noticeable and significant difference was observed after exposure to AuS0302-RIT. The number of AuS0302-RIT nanoparticles internalized in A549 was two-fold higher compared to NCIH441 cells. As demonstrated in Figure 4 B, the data of internalized AuS0302-RIT nanoparticles compared to the initial concentration emphasizes this difference while A549 and H441 cells internalized 36% and 19% of total available gold nanoparticles, respectively. In summary, the epithelial cells internalized a higher amount of gold nanoparticles than the endothelial cell types after 24 hours. In addition, the size of the nanoparticles influenced the amount of internalized gold nanoparticles. The epithelial cells internalized a higher amount of smaller gold nanoparticles (−RIT and -RIS02) while HDMEC showed a higher affinity to the 25 nm-sized particles (−RIS04) compared to the smaller gold nanoparticles. However, in these studies gold nanoparticles were internalized in very low amounts by brain endothelial cells. Interestingly, the amount of sodium citrate on the particle surfaces did not influence the uptake into cells.

## Discussion

Uboldi et al. have recently demonstrated that AuNPs induced cytotoxicity in the AT-II-like human epithelial cells, A549 and H441, and the effects exerted by gold nanoparticles (AuNPs) correlated with the presence of sodium citrate on the particle surface [10]. In this study we wished to determine if the same effect would be observed in human endothelial cells after exposure to the same gold nanoparticles. AuS0302-RIT, AuS0302-RIS02 and AuS0302-RIS04, which have sizes of 10 nm, 11 nm and 25 nm respectively, as well as different amounts of sodium citrate on their surface, were studied with respect to their potential cytotoxicity and their uptake behavior.

We found that only high concentrations of AuNPs (500 μM and 1000 μM) negatively influence the cell viability in both endothelial cell types. The significant increase in cell viability after the treatment with 500 μM and 1000 μM AuS0302-RIT is not fully understood but may be explained by a higher mitochondrial activity of cells incubated with up to 1000 μM gold nanoparticles or a very slight cross-reaction of gold nanoparticles with the substrate of the MTS assay. In addition, a small but not significant increase in cell viability of hCMEC and NCIH441 could also be observed [10]. Thus, to exclude misinterpretation of the data three different assays (MTS, LDH, and proliferation experiments) were performed to determine the effects on the cell viability. In summary, a positive effect on cell viability after treatment with 500–1000 μM AuS0302-RIT cannot be determined. Furthermore a decrease in proliferation rate as measured by an assay for the proliferation factor Ki-67 [19-21], was higher in primary human dermal microvascular endothelial cells (HDMEC) compared to the human cerebral microvascular endothelial cell line, (hCMEC/D3). Even 50 μM AuS0302-RIS04 decreased the proliferation rate in HDMEC while the proliferation in hCMEC exposed to this concentration was not affected. The higher amount of internalized AuS0302-RIS04 might be the reason for the decrease of cell proliferation of HDMEC compared to hCMEC after exposure to 50 μM gold nanoparticles. Dyer and Patterson showed that numerous properties and characteristics of endothelial cells from various locations of the body differ [22]. These variations (i.e. permeability, surface receptors) might generally explain the reduced proliferation rate (here determined by Ki67 expression) in HDMEC after exposure to 50 μM AuS0302-RIS04. In addition to that the increased particle concentration within the cells might decrease the motility of the cells and thus might impair the cell growth and the proliferation. Mironava et al. has previously shown that increased uptake of nanoparticles in human dermal fibroblast was accompanied by a higher amount of vesicles within the cells which impaired the cytoskeleton and influenced cell division [23]. However an overload of the endothelial cells with gold nanoparticles could not be observed using transmission electron microscopy (TEM). In addition, cell cycle arrest might also be an explanation for decreased proliferation. It has previously shown that human prostate cancer cells (DU-145) arrested in G2/M phase of the cell cycle after exposure to AuNPs [24]. In general, this arrest was shown to be accompanied by increased apoptosis [25,26]. However, after treatment with 50 μM of gold nanoparticles no increase of cell death (measured by LDH-assay) was determined in our investigations. Only much higher concentrations of gold nanoparticles (>500 μM) resulted in an increased cell death in both endothelial cell types. These observations might be explained by the arrest in G2/M phase. Since AuS0302-RIS02 (11 nm) and AuS0302–RIS04 (25 nm) but not AuS0302-RIT (10 nm) induced these effects, it could be concluded that the size of the AuNPs was not a significant factor, but that the higher amount of sodium citrate on the particle surface was the cause for the increase of cytotoxicity. Certainly, the higher concentrations of gold nanoparticles (≥500 μM) resulted in a decreased proliferation rate of both endothelial cells even after exposure to AuS0302-RIT. However the effects on proliferation by particles with a high amount of sodium citrate were higher compared to the effects caused by AuS0302-RIT. In general, citrate-stabilized gold nanoparticles may build agglomerates in cell culture media with serum proteins. So, not only single gold nanoparticles but potential agglomerates come into contact with cells. Due to the different citrate concentrations of the particles different amounts of agglomerates may be built and various impacts on cells may be the result. This may explain the effect of the gold nanoparticles with higher citrate concentration on all cell types. However, the group around Gosens has previously shown that the effects of single and agglomerated citrate-stabilized gold nanoparticles on lung cells were not altered [27]. Thus, the effects of agglomerates can be neglected. Beside this, other factors may be responsible and a detailed clarification of subcellular events regarding the exact nature of the toxic effects (apoptosis, necrosis) and the impairment of the cell proliferation needs to be done. In general, the physico-chemical properties of nanoparticles (i.e. surface modifications) influence the interaction of particles with cells and the effects related to these interactions [28].

In addition to the surface modification, it has been demonstrated by different groups that the size of gold nanoparticles has an influence on their internalization and the subsequent cellular reaction, such as cytotoxicity [29]. Although only two different-sized gold nanoparticles (10-11 nm and 25 nm) were examined in the present studies, an obvious size effect was not correlated to cytotoxic effect when endothelial cells were exposed to these nanoparticles. Furthermore, differences with two nanoparticles of the same size could also be observed. Both AuS0302-RIT and AuS0302RIS02 were the same size, however, a decrease in the expression of the proliferation factor Ki-67 as well as the increased toxicity was observed after exposure of cells to 1000 μM AuS0302-RIS02, indicating that size was not- but that the concentration of citrate was the relevant factor for causing these effects. In addition, the same effects were detected after the exposure of the endothelial cells to AuS0302-RIS04 which contained the same amount of citrate but were a different size. These results were also observed by Uboldi et al. who demonstrated that it was not the size but the amount of sodium citrate present in the gold nanoparticles that were responsible for the cytotoxic effects on the lung epithelial cell lines, A549 and NCIH441 [10].

Interestingly, the effects exerted on the proliferation rate of A549 and NCIH441 after exposure to 500 μM to 1000 μM AuNPs were more severe compared to those observed on the endothelial cells examined in the present study with the same nanoparticles [10]. The proliferation rate of epithelial cells was decreased compared to endothelial cells after exposure to high concentrations of gold nanoparticles with a higher concentration of sodium citrate on their surface. Moreover, the cytotoxic effects and the effects on cell viability were milder in endothelial than in epithelial cells as measured by the LDH and MTS assays. These studies indicate that specific cell types respond differently to a particular nanoparticle.

Further studies were carried out to determine whether a correlation existed between the amount of internalized particles and cytotoxicity. Cells examined by transmission electron microscopy showed that the AuNPs were internalized by both epithelial and endothelial cells. In previous studies, it was shown that in the epithelial cells A549 and NCIH441 the internalized AuNPs were located in vesicles enclosed in the perinuclear region [10]. The same results were observed in the human endothelial cells examined in these studies. Others, in contrast to these findings, demonstrated by TEM analyses that 25 nm-sized gold nanoparticles could be detected in a freely dispersed form in the cytoplasm of airway epithelial cells A549 [30]. In addition to the intracellular localization of the AuNPs the TEM analyses showed that the exposure to gold nanoparticles did not alter the cell morphology.

A quantification of internalized AuNPs was carried out by the more sensitive atomic emission spectroscopy (ICP-AES). These analytical results obtained after analysis of cell populations exposed to nanoparticles corresponded with the TEM images of single cells, namely the presence of sodium citrate did not influence the uptake behavior of the gold nanoparticles in endothelial and epithelial cells. Similar amounts of gold nanoparticles were taken up regardless of the presence or absence of citrate. In addition, ICP-AES analysis showed that epithelial cells internalized more gold nanoparticles than endothelial cells. Similar results were reported by Xia et al. who also showed that different uptake mechanisms of cationic polystyrene nanospheres were observed in human endothelial cells (HMEC) compared to epithelial cells (BEAS-2B). However, these results were not correlated to any quantitative parameter of internalization of the nanospheres. Nevertheless, BEAS-2B cells reacted in a more sensitive manner compared to endothelial cells and exhibited necrotic cell death after exposure. These findings were also observed in RAW 264.7 murine macrophages, with the authors concluding that this difference was due to the more sensitive character of epithelial cells as the first-line-of-defense-features in pulmonary cell types [31]. In our study, the more sensitive character of the epithelial cells after exposure to AuS0302-nanoparticles was reflected by a decreased proliferation rate demonstrated by the Ki-67 assay, and not increased cytotoxicity after exposure to the AuNPs as reported by the group of Xia. The higher sensitivity could also not be explained in detail by Xia et al. One possible explanation for the difference in the uptake between epithelial and endothelial cells might be a different interaction of nanoparticles with specific domains of the plasma membrane. According to Nel et al. different forces are present between the particle and the cell surface which resist or promote nanoparticle wrapping [28]. Both promotive and resistive forces, which might differ from one cell type to another, could influence the uptake of nanoparticles. This hypothesis needs to be investigated further but could be an explanation for the different quantities of gold nanoparticles measured within the various cell types.

The more severe effect on the proliferation of epithelial cells compared to endothelial cells could also be explained by the higher number of gold nanoparticles internalized, in combination with the presence of sodium citrate on the particle surface. AuS0302-RIT nanoparticles were internalized in high amounts after 24 hours exposure, while the proliferation rate in epithelial cells was only slightly decreased compared to AuS0302-RIS02 and AuS0302-RIS04 even after 48 hours. Nevertheless, the influence of sodium citrate on the surface of particles not internalized could also play a role in inducing toxic effects on the cells.

## Conclusions

In conclusion the higher amount of citrate on the particle surface resulted in a higher impairment of cell viability, but did not enhance or reduce the uptake behavior in endothelial or epithelial cells. In addition, epithelial and endothelial cells exhibited different uptake behaviors for citrate-stabilized gold nanoparticles, which might be related to different interactions occurring at the nanoparticle-cell-surface interface. The different uptake in epithelial cells might explain the higher reduction of proliferation of these cells after exposure to AuNPs treatment although more detailed investigations are necessary to determine subcellular events. Nevertheless an extrinsic effect of sodium-citrate stabilized particles could not be excluded. Thus, the amount of sodium citrate should be reduced to a level on which the stability of the particles and the safety for biomedical applications are guaranteed.

## Methods

### Gold nanoparticles

The water dispersible gold nanospheres were synthesized and characterized as described previously [10]. Briefly, the size of AuS0302-RIT and AuS0302-RIS02 was measured by scanning transmission electron microscopy (STEM) and was determined to be 10 nm and 11 nm, respectively, while the size of AuS0302-RIS04 was 25 nm. The gold nanoparticles also differed in the amount of sodium citrate on the particle surface. AuS0302-RIT contained 7% of sodium citrate compared to AuS0302-RIS02 and AuS0302-RIS04. A reduction of sodium citrate did not result in an agglomeration or aggregation of the particles as indicated by the same polydispersity index (PDI) in the different NP suspensions. The data is summarized in Table 1.

**Table 1:** **Physicochemical characterization of the gold nanoparticles (partially reprinted from Uboldi et al.**[10]**)**

**Samples**	**%Au w/w**	**% citrate w/w**	**PDI**	**dV mean (nm)**	**D STEM (nm)**
**AuS0302-RIT**	0.256	0.048	0.33	21.2	9.8
**AuS0302-RIS02**	0.282	0.686	0.33	27.2	11.2
**AuS0302-RIS04**	0.288	0.686	0.36	28.2	25

PDI: polydispersity index.

dV: mean diameter calculated by dynamic light scattering (DLS).

D STEM: scanning transmission electron micrographs.

### Isolation of cells and cell culture

Human dermal microvascular endothelial (HDMEC) cells were isolated from juvenile foreskin as previously described [32]. Briefly, cells were isolated by cutting the foreskin into small pieces and after enzymatic digestion in 0.4% collagenase (Gibco, Carlsbad, USA) for 16 hours the epidermis was manually separated from the dermis. After a second incubation with versene (Gibco, Carlsbad, USA) and 80 μl 2.5% trypsin (Gibco, Carlsbad, USA) for 2 hours, and after a mechanical treatment of the foreskin and filtering of the digested tissue, the cells were resuspended, seeded onto 0.2% gelatin–coated culture flasks and cultured in Endothelial cell basal medium (ECBM; Customer Formulation) supplemented with supplement mix (PromoCell, Heidelberg, Germany) and 10000 units/ml penicillin (Gibco, Carlsbad, USA)/10000 μg/ml streptomycin (Gibco, Carlsbad, USA). To separate the endothelial cells from other contaminating cells such as fibroblasts, two separating steps with magnetic CD-31 beads were performed after the cells were confluent. Following the first separating step cells were grown in ECBM supplemented with 15% fetal bovine serum, 2.5 ng/ml basal fibroblast growth factor and 10 μg/ml sodium heparin (Sigma-Aldrich, St. Luis, USA), and 10000 units/ml penicillin (Gibco, Carlsbad, USA)/10000 μg/ml streptomycin (Gibco, Carlsbad, USA). Cells were only used up to passage three.

The human cerebral microvascular endothelial cell line hcMEC/D3 was provided from the group of Pierre-Olivier Couraud (Department of Cell Biology, Institut Cochin, Paris, France). The morphological characteristics and the expression of numerous typical endothelial markers and cell adhesion molecules were previously shown [33], and were also confirmed by our group after culturing the cells on fibronectin-coated culture flasks in ECBM complete culture medium. The cells were sub-cultivated twice a week.

The human alveolar type-II (AT-II)-like cell lines A549 (ATCC number CCL-185,) and NCIH441 (ATCC number HTB-174) were purchased by LGC Promochem (Wesel, Germany). Cells were cultured in complete cell culture medium composed of RPMI 1640 with L-Glutamine (Invitrogen Corporation, Germany) and supplemented with 10% fetal bovine serum (Sigma Aldrich, St. Luis, USA) and penicillin (10000U/ml) and streptomycin (10000 μg/ml) (Invitrogen Corporation, Germany). Cells were passaged weekly and maintained under standard conditions (5% CO_2_ and 95% humidified condition at 37°C).

### Cell viability, cell proliferation and cytotoxicity assay

The cytotoxicity induced by gold nanoparticles was determined by the MTS assay, the Ki-67 assay and the lactate dehydrogenase (LDH) release assay. Cells were seeded in 96-well plates (TPP, Switzerland, 0.33 cm² growth area) and exposed for 48 hours to 100 μl of different concentrations of gold nanoparticles ranging from 0 μM to 1000 μM. Nanoparticles were dispersed in complete cell culture medium in ECBM culture medium before being added to HDMEC and hCMEC/D3. Three to four independent experiments and three replicates for each experiment were performed.

Cell viability was measured using the CellTiter 96 AQ_ueous_ non-radioactive assay (Promega, Mannheim, Germany). After 48 hours incubation with gold nanoparticles the supernatant was collected in a 96-well plate to analyze the LDH release. Cells were washed with 0.2% bovine serum albumin in HEPES buffer (pH 7.2) and incubated with cell culture medium containing 20% MTS solution at 37°C. The medium was transferred to a new 96-well and the absorption was measured by spectroscopy at a wavelength of λ = 490 nm. The absorption of the untreated control cells was set as 100% viability.

The cells were fixed with methanol/ethanol solution (2:1) at room temperature for 20 minutes. Afterwards cells were washed in PBS and permeabilized with 0.1% Triton-X-100 in PBS for 10 minutes. After additional washing steps with a solution containing PBS/0.05% Tween-20, the cells were incubated (45 minutes at 37°C on a rotating plate) with 1 μg/ml mouse anti-human Ki-67 antibody (clone MIB-1; DAKO, Germany). Cells were then incubated for 45 minutes at 37°C with anti-mouse IgG1 peroxidase (DAKO, Germany), and washed with PBS/0.05% Tween-20. Subsequently, cells were incubated for 10–20 minutes at 37°C with a solution (170 μl/well) composed of 50 mL citric buffer mixed with 20 μl 30% oxygen peroxide and o-phenylenediamine. After the incubation the enzymatic reaction was stopped by transferring the solution to a new 96-well plate containing 3 M hydrochloric acid (HCl) (50 μl/well). The absorption was detected by spectrophotometry at a wavelength of λ = 492 nm. Results are depicted as mean values in% of the cellular proliferation of the untreated controls. LDH release into the medium was detected using the CytoTox 96 non-radioactive cytotoxicity assay (Promega, Mannheim, Germany) as recommended by the manufacturer. The release of LDH in the untreated cells was used as control, and the LDH activity of lysed cells set at 100%. Data were analyzed using GraphPad Prism version 5.00 for Windows (GraphPad Software, San Diego California USA, http://www.graphpad.com).

### Uptake and transmission electron microscopy studies

Thermanox cover slips (NUNC–Thermo Fisher Scientific, Germany) were transferred into a 24-well plate (TPP, Switzerland, 2.0 cm² growth area) and were coated with fibronectin. Cells were seeded on the cover slips and cultivated until confluence. For the incubation at 37°C, cells were exposed to 1.1 ml 300 μM gold nanoparticles AuS0302-RIT, AuS0302-RIS02 and AuS0302-RIS04. After 24 hours of exposure cells were washed and then fixed with 2.5% glutaraldehyde in cacodylate buffer (pH 7.2) for 20 min. This was followed by a fixation step in 1% (w/v) osmium tetroxide for 2 hours and dehydration in ethanol. Cells were transferred through propylene oxide. Afterwards the samples were embedded in agar-100 resin (PLANO, Germany) and polymerized at 60°C for 48 hours. Ultrathin sections were cut with an ultramicrotome (Leica Microsystems, Germany), placed onto copper grids and stained with 1% (w/v) uranyl acetate in alcoholic solution and lead citrate. Ultrastructural analysis was performed with a transmission electron microscope EM 410 (Philips; Eindhoven, Netherlands).

### Quantification of internalized Au-NPs

Cells were seeded onto fibronectin-coated 24-well plates (TTP, Switzerland, 2.0 cm² growth area). After reaching confluence the medium was replaced by the 1.1 ml nanoparticle suspension (50 μM). After treatment for 24 h the cells were washed with HEPES + 0.2% BSA, detached by trypsin incubation and transferred after the addition of 0.9 ml PBS to microcentrifuge tubes. The cell suspension was stored at −20°C until analysis. To the cell lysate solution 0.15 ml of aqua regia (3:1 hydrochloric acid : nitric acid [Caution must be taken when handling, as reactions are exothermic]) was added. Following incubation overnight, the samples were then further diluted to 5 mL using milliQ water to give a total sample volume of 5 ml. These samples were then analysed for total gold content by inductively coupled plasma atomic emission spectroscopy (ICP-AES), and the measurement was repeated 3 times for each sample. A PerkinElmer Optima 5300 DV was used, and values reported were based on a calibration curve using an Au ICP standard from Sigma-Aldrich (UK). Since the diameter of the gold nanoparticles was known we calculated the gold atoms per particle and determined the number of particles per well. To determine the percentage share of particles internalized by the cells we used the total amount of gold nanoparticles calculated by the concentration applied, the diameter of particles and the number of particles internalized.

## Competing interests

The authors declare that they have no competing interests.

## Authors' contributions

CF: contributed by planning and performing the experiments, analysing and interpreting the results and by preparing the manuscript. CU has contributed by interpreting the results and critically revising the manuscript. MIG has performed the ICP-AES analyses and summarized the results. REU and CJK contributed to the scientific planning and interpretation of data and were involved in the manuscript revision. BBW, IAR and POC have created the immortalized cell line of normal human brain endothelial cells, termed hCMEC/D3, and were involved in the manuscript revision. All authors read and approved the final manuscripts.

## Supplementary Material

Additional file 1 **Internalization of gold nanoparticles in A549 and NCIH441 analyzed by transmission electron microscopy.** A549 (A-C) and NCI-H441 (D-F) were incubated with 300 μM gold nanoparticles at 37°C for 24 hours. Cells were extensively washed, fixed and examined by transmission electron microscopy (TEM). All gold nanoparticles are found in intracellular vesicles which were mostly located in the perinuclear region. None of the nanoparticles can be found in the nuclei. The arrow heads indicate the gold nanoparticles within the vesicles. Click here for file

## References

[B1] Sperling RA Gil P Zhang F Zanella M Parak WJ Biological applications of gold nanoparticles Chem Soc Rev 2008 37 9 1896 1908 10.1039/b712170a 18762838

[B2] Boisselier E Astruc D Gold nanoparticles in nanomedicine: preparation, imaging, diagnostics, therapies and toxitiy Chem Soc Rev 2009 38 6 1759 1782 10.1039/b806051g 19587967

[B3] Sopjani M Föller M Lang F Gold stimulates Ca2+ entry into and subsequent suicidal death of erythrocytes Toxicology 2008 244 271 279 10.1016/j.tox.2007.12.001 18207621

[B4] Sereemaspun A Rojanathanes R Wiwanitkit V Effect of gold nanoparticle on renal cell: an implication for exposure risk Renal failure 2008 30 323 325 10.1080/08860220701860914 18350452

[B5] Cho W-S Cho M Jeong J Choi M Cho H-Y Han BS Kim SH Kim HO Lim YT Chung BH Jeong J Acute toxicity and pharmacokinetics of 13 nm-sized PEG-coated gold nanoparticles Toxicology and Applied Pharmacology 2009 236 1 16 24 10.1016/j.taap.2008.12.023 19162059

[B6] Gibson MI Danial M Klok H-A Sequentially Modified, Polymer-Stabilized Gold Nanoparticle Libraries: Convergent Synthesis and Aggregation Behavior ACS Combinatorial Science 2011 13 3 286 297 10.1021/co100099r 21384914

[B7] Pan Y Leifert A Ruau D Neuss S Bornemann J Schmid G Brandau W Simon U Jahnen-Dechent W Gold nanoparticles of diameter 1.4 nm trigger necrosis by oxidative stress and mitochondrial damage Small (Weinheim an der Bergstrasse, Germany) 2009 5 2067 2076 10.1002/smll.200900466 19642089

[B8] Arvizo RR Miranda OR Thompson MA Pabelick CM Bhattacharya R Robertson JD Rotello VM Prakash YS Mukherjee P Effect of nanoparticle surface charge at the plasma membrane and beyond Nano letters 2010 10 2543 2548 10.1021/nl101140t 20533851 PMC2925219

[B9] Hauck TS Ghazani AA Chan WCW Assessing the effect of surface chemistry on gold nanorod uptake, toxicity, and gene expression in mammalian cells Small (Weinheim an der Bergstrasse, Germany) 2008 4 153 159 10.1002/smll.200700217 18081130

[B10] Uboldi C Bonacchi D Lorenzi G Hermanns MI Pohl C Baldi G Unger RE Kirkpatrick CJ Gold nanoparticles induce cytotoxicity in the alveolar type-II cell lines A549 and NCIH441 Part Fibre Toxicol 2009 6 18 10.1186/1743-8977-6-18 19545423 PMC2705341

[B11] Cines DB Pollak ES Buck CA Loscalzo J Zimmerman GA McEver RP Pober JS Wick TM Konkle BA Schwartz BS Endothelial cells in physiology and in the pathophysiology of vascular disorders Blood 1998 91 3527 3561 9572988

[B12] Kirkpatrick CJ Bittinger F Klein CL Hauptmann S Klosterhalfen B The role of the microcirculation in multiple organ dysfunction syndrome (MODS): a review and perspective Virchows Arch 1996 427 461 476 8624575 10.1007/BF00199506

[B13] Kunzmann A Andersson B Thurnherr T Krug H Scheynius A Fadeel B Toxicology of engineered nanomaterials: Focus on biocompatibility, biodistribution and biodegradation Biochimica et biophysica acta 2010 1810 3 361 73 20435096 10.1016/j.bbagen.2010.04.007

[B14] Bartczak D Sanchez-Elsner T Louafi F Millar TM Kanaras AG Receptor-Mediated Interactions between Colloidal Gold Nanoparticles and Human Umbilical Vein Endothelial Cells Small 2010 7 3 388 94 21294268 10.1002/smll.201001816

[B15] Bartczak D Muskens OL Nitti S Sanchez-Elsner T Millar TM Kanaras AG Interactions of Human Endothelial Cells with Gold Nanoparticles of Different Morphologies Small 2012 8 122 130 10.1002/smll.201101422 22102541

[B16] Chithrani BD Ghazani AA Chan WCW Determining the Size and Shape Dependence of Gold Nanoparticle Uptake into Mammalian Cells Nano Letters 2006 6 662 668 10.1021/nl052396o 16608261

[B17] Ma Y-J Gu H-C Study on the endocytosis and the internalization mechanism of aminosilane-coated Fe3O4 nanoparticles in vitro Journal of materials science Materials in medicine 2007 18 2145 2149 10.1007/s10856-007-3015-8 17665123

[B18] Nativo P Prior IA Brust M Uptake and Intracellular Fate of Surface-Modified Gold Nanoparticles ACS Nano 2008 2 1639 1644 10.1021/nn800330a 19206367

[B19] Scholzen T Gerdes J The Ki-67 protein: from the known and the unknown Journal of cellular physiology 2000 182 311 322 10.1002/(SICI)1097-4652(200003)182:3<311::AID-JCP1>3.0.CO;2-9 10653597

[B20] Gerdes J Lemke H Baisch H Wacker HH Schwab U Stein H Cell cycle analysis of a cell proliferation-associated human nuclear antigen defined by the monoclonal antibody Ki-67 Journal of immunology (Baltimore, Md : 1950) 1984 133 1710 1715 6206131

[B21] Sasaki K Murakami T Kawasaki M Takahashi M The cell cycle associated change of the Ki-67 reactive nuclear antigen expression Journal of cellular physiology 1987 133 579 584 10.1002/jcp.1041330321 3121642

[B22] Dyer LA Patterson C Development of the endothelium: an emphasis on heterogeneity Seminars in thrombosis and hemostasis 2010 36 227 235 10.1055/s-0030-1253446 20490975 PMC3328212

[B23] Mironava T Hadjiargyrou M Simon M Jurukovski V Rafailovich MH Gold nanoparticles cellular toxicity and recovery: Effect of size, concentration and exposure time: Nanotoxicology Nanotoxicology 2010 4 120 137 10.3109/17435390903471463 20795906

[B24] Roa W Zhang X Guo L Shaw A Hu X Xiong Y Gulavita S Patel S Sun X Chen J Gold nanoparticle sensitize radiotherapy of prostate cancer cells by regulation of the cell cycle Nanotechnology 2009 20 375101 10.1088/0957-4484/20/37/375101 19706948

[B25] Fang JL Beland FA Long-term exposure to zidovudine delays cell cycle progression, induces apoptosis, and decreases telomerase activity in human hepatocytes Toxicol Sci 2009 111 120 130 10.1093/toxsci/kfp136 19541796 PMC2734309

[B26] Zhang L Zhang J Hu C Cao J Zhou X Hu Y He Q Yang B Efficient activation of p53 pathway in A549 cells exposed to L2, a novel compound targeting p53-MDM2 interaction Anticancer Drugs 2009 20 416 424 10.1097/CAD.0b013e32832aa7b0 19579266

[B27] Gosens I Post JA de la Fonteyne LJ Jansen EH Geus JW Cassee FR de Jong WH Impact of agglomeration state of nano- and submicron sized gold particles on pulmonary inflammation Part Fibre Toxicol 2010 7 37 10.1186/1743-8977-7-37 21126342 PMC3014867

[B28] Nel AE Madler L Velegol D Xia T Hoek EMV Somasundaran P Klaessig F Castranova V Thompson M Understanding biophysicochemical interactions at the nano-bio interface Nat Mater 2009 8 543 557 10.1038/nmat2442 19525947

[B29] Pan Y Neuss S Leifert A Fischler M Wen F Simon U Schmid G Brandau W Jahnen-Dechent W Size-Dependent Cytotoxicity of Gold Nanoparticles Small 2007 3 1941 1949 10.1002/smll.200700378 17963284

[B30] Rothen-Rutishauser B Mühlfeld C Blank F Musso C Gehr P Translocation of particles and inflammatory responses after exposure to fine particles and nanoparticles in an epithelial airway model Part Fibre Toxicol 2007 4 9 10.1186/1743-8977-4-9 17894871 PMC2039730

[B31] Xia T Kovochich M Liong M Zink JI Nel AE Cationic Polystyrene Nanosphere Toxicity Depends on Cell-Specific Endocytic and Mitochondrial Injury Pathways ACS Nano 2008 2 85 96 10.1021/nn700256c 19206551

[B32] Unger RE Krump-Konvalinkova V Peters K Kirkpatrick CJ In Vitro Expression of the Endothelial Phenotype: Comparative Study of Primary Isolated Cells and Cell Lines, Including the Novel Cell Line HPMEC-ST1.6R Microvascular Research 2002 64 384 397 10.1006/mvre.2002.2434 12453433

[B33] Weksler BB Subileau EA Perrière N Charneau P Holloway K Leveque M Tricoire-Leignel H Nicotra A Bourdoulous S Turowski P Blood–brain barrier-specific properties of a human adult brain endothelial cell line The FASEB journal : official publication of the Federation of American Societies for Experimental Biology 2005 19 1872 1874 10.1096/fj.04-3458fje 16141364

