# Supplementary figures and images for: Uptake and cytotoxicity of citrate-coated gold nanospheres: Comparative studies on human endothelial and epithelial cells
Source: Part Fibre Toxicol. 2012 Jul 3;9:23. doi: 10.1186/1743-8977-9-23 (PMC3407003; doi:10.1186/1743-8977-9-23)

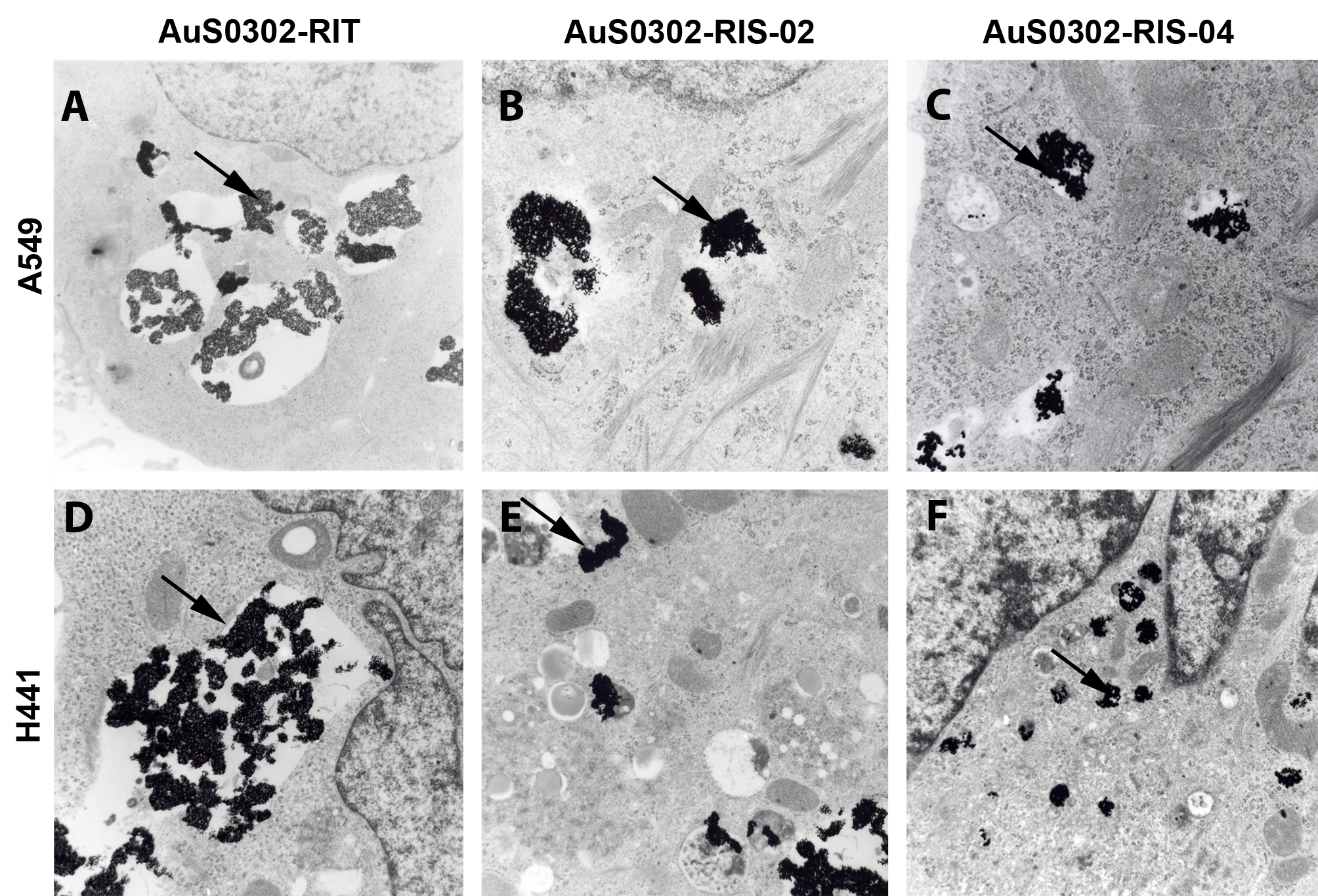

Supplement: Additional file 1 — Internalization of gold nanoparticles in A549 and NCIH441 analyzed by transmission electron microscopy. A549 (A-C) and NCI-H441 (D-F) were incubated with 300 μM gold nanoparticles at 37°C for 24 hours. Cells were extensively washed, fixed and examined by transmission electron microscopy (TEM). All gold nanoparticles are found in intracellular vesicles which were mostly located in the perinuclear region. None of the nanoparticles can be found in the nuclei. The arrow heads indicate the gold nanoparticles within the vesicles. [file 1743-8977-9-23-S1.tiff]
